# Ovarian Teratoma-Related Paraneoplastic Neurological Syndromes

**DOI:** 10.3389/fonc.2022.892539

**Published:** 2022-05-16

**Authors:** Jingfang Lin, Minjin Wang, Jierui Wang, Jinmei Li

**Affiliations:** ^1^ Department of Neurology, West China Hospital, Sichuan University, Chengdu, China; ^2^ Department of Laboratory Medicine, West China Hospital, Sichuan University, Chengdu, China

**Keywords:** ovarian teratoma, paraneoplastic neurological syndromes, pathological findings, antibodies, anti-N-methyl-D-aspartate receptor encephalitis

## Abstract

Paraneoplastic neurological syndromes (PNSs) are a group of neurological disorders triggered by an underlying remote tumor. Ovarian teratoma (OT) is the most common histologic type of germ cell tumor in females. The most common PNSs associated with OT is anti-N-methyl-D-aspartate receptor (NMDAR) encephalitis. However, with the increasing number of new antibodies reported over the last decade, the clinical spectrum of OT-related PNSs is also expanding. Our knowledge of OT-related PNSs is still far from complete. Here, we provide a comprehensive review of the most recent findings in the field of OT-related PNSs, with a particular focus on their clinical and pathological characteristics. Overall, the description of neuronal antibodies in PNSs associated with OT strongly suggests that antibodies may be responsible for the clinical symptoms in some cases. OT-related PNSs are associated with various clinical manifestations, including anti-NMDAR encephalitis, limbic encephalitis, encephalomyelitis, progressive cerebellar syndrome and opsoclonus-myoclonus syndrome. The pathological characteristics of the OT suggest that the mechanism of PNSs is probably due to heteromorphic neurons in the tumor tissue, the ectopic expression of the antigens in neural tissue within the teratomas and patients’ unusual immune response. Despite the severity of the neurological syndromes, most patients with OT-related PNSs showed good neurologic response to early tumor resection combined with immunotherapy. To further advance the management of OT-related PNSs, additional studies are needed to explore this complex topic.

## Introduction

Paraneoplastic neurological syndromes (PNSs) are a group of neurological disorders triggered by an underlying remote tumor but not directly caused by cancer metastasis (1). PNSs usually develop before the diagnosis of the tumor. According to the recently updated diagnostic criteria for PNSs, PNSs mainly include the following clinical manifestations: encephalomyelitis, limbic encephalitis (LE), rapidly progressive cerebellar syndrome (PCD), opsoclonus-myoclonus syndrome (OMS), sensory neuronopathy, gastrointestinal pseudo-obstruction (enteric neuropathy), and Lambert-Eaton myasthenic syndrome (LEMS) (2). At present, small-cell lung cancer, breast cancer, testicular cancer, and lymphoma have all been reported as possible causes of PNSs. Ovarian teratoma (OT), which accounts for approximately 20% of all ovarian neoplasms, is one of the common tumor types causing PNSs (3, 4). Anti-N-methyl-D-aspartate receptor (NMDAR) encephalitis is a well-known PNSs associated with OT (5). However, with the increasing number of new antibodies reported over the last decade, the clinical spectrum of OT-related PNSs is expanding. For example, recent studies suggest that OT may trigger paraneoplastic neuromyelitis optica spectrum disorders (NMOSD), myelin oligodendrocyte glycoprotein antibody-associated encephalomyelitis (MOG-EM), and anti-contactin-associated protein-like 2 (CASPR2) antibody-associated autoimmune encephalitis (6–9). Our knowledge of OT-related PNSs is still far from complete. The paucity of scientific understanding of OT-related PNSs results in delayed diagnosis and decision-making difficulties, creating a vicious cycle. In the present review, we describe OT-related PNSs as comprehensively as possible, focusing on the clinical spectrum and distinctive immunopathology of teratoma. Due to the low incidence of OT-associated PNSs, the current literatures are mostly case reports or case series studies and mainly focuses on anti-NMDAR encephalitis. These data will expand clinicians’ awareness about OT-related PNSs and allow them to perform the OT screening in appropriate patients, leading to early recognition of this condition, preventing more disability, and increasing further recovery.

## Frequency and Classification of Ovarian Teratoma-Related PNSs

The frequency of OT-related PNSs lacks objective and comprehensive documentation. To date, there have been only two reports showing the incidence rate of anti-NMDAR encephalitis in patients with ovarian teratoma. A Japanese single-center retrospective study, involving 343 patients from January 2008 to December 2016, found anti-NMDAR encephalitis in only 6 (1.17%) of all ovarian teratoma patients (10). The second one was retrospectively identified in a series of 233 Israeli patients, which found that anti-NMDAR encephalitis was diagnosed in 0.85% of women with mature teratomas (MTs) over 12 years (11). However, neither of the above publications analyzed PNSs other than anti-NMDAR encephalitis nor conducted comprehensive antibody screening.

Comparatively, three studies investigated whether neurologically asymptomatic individuals diagnosed with OT may have positive serum autoimmune antibodies. Two of the studies (recruited 10 German OT patients and 80 Chinese OT patients) aimed at detecting NMDAR antibodies showed that NMDAR antibodies were not detected in the serum of all neurologically asymptomatic patients with OT (12, 13). Another study recruiting 20 patients with OT conducted more extensive antibody testing (including (NMDAR-NR1a, NMDAR-NR1a/NR2b, a-amino-3-hydroxy-5-methyl-4-isoxazolepropionic acid receptor [AMPAR]-GluR1/GluR2, DPPX-IF1, DPPX-IF2, γ-aminobutyric acid receptor-B [GABAR]-B1/B2, leucine-rich glioma-inactivated protein 1 [LGI1], CASPR2, GLRA1b, GRM1, GRM5, MOG, Tr/DNER, anti-aquaporin-4 [AQP4], GAD65, GAD67, ZIC, ARHGAP26, Yo, amphiphysin, Hu, Ri, Ma1, Ma2, CV2, Sox-1, and recoverin) and found that none of the patients with OT had autoimmune antibodies (14). These data indicate that serum NMDAR antibody testing can be omitted in patients with OT unless complicating autoimmune encephalitis is suspected. However, due to the small samples included or the single type of antibodies tested in these studies, we need to be more cautious about these conclusions.

## The Neurological Syndromes of OT-Related PNSs

### Autoimmune Encephalitis (AE)

Based on previous studies, OT exhibited few gynecological symptoms (lower abdominal pain before the onset of acute torsion of ovarian teratoma) in patients with PNSs. The confirmation of OT is usually detected during further examination because of the combined PNSs.

**Figure 1:**
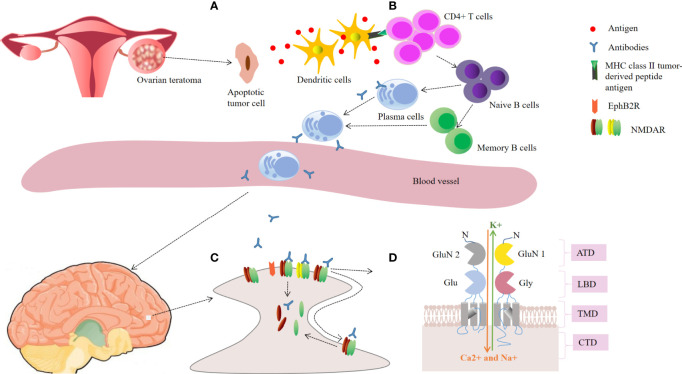
Schematic representation of the possible pathogenesis in ovarian teratoma-related anti-NMDAR encephalitis. **(A)** Ovarian teratoma contains apoptotic tumor cells, neuroglial cells and inflammatory infiltrates. Antigens of N-methyl-d-aspartate receptor (NMDAR) released by apoptotic tumor cells are loaded into dendritic cells; **(B)** The antigen is presented to naive B cells by mature dendritic cells in cooperation with CD4 T cells, leading to generation of plasma cells and memory B cells. Plasma cells migrate into the brain and generate large amounts of IgG autoantibodies; **(C)** The autoantibodies lead to receptor cross-linking and internalization, and disrupting the interaction between NMDAR and Ephrin-B2 receptor (EphB2R), thus reducing the number of NMDARs on the neuronal surface; **(D)** The GluN1 and GluN2 subunits of the NMDAR. Antibodies pre-dominantly bind to an epitope region in the amino-terminal domain (ATD) of GluN1. CTD, carboxyl-terminal domain; LBD, ligand binding domain; TMD, transmembrane domain.

The most common PNSs caused by OT is anti-NMDAR encephalitis (**Table 1**) (10, 15–39), which is an autoimmune neurological disease characterized by a clinical presentation of encephalitis and the presence of cerebrospinal fluid (CSF) antibodies against the GluN1 subunit of the NMDAR (15). The prevailing theory for the pathogenesis of anti-NMDAR encephalitis is that antibody-mediated injury to neurons is caused by antibodies generated against NMDARs on the teratoma that cross the blood brain barrier (**Figure 1**) (49, 50). It has been reported that OTs exist in almost 37.4% of cases with anti-NMDAR encephalitis, primarily in young females aged between 18 and 35 years (51). Some patients with anti-NMDAR encephalitis manifest prodromal symptoms, usually including fever and headache (28). In 2016, a team of experts proposed the following guidelines for the diagnosis of definite anti-NMDAR encephalitis. It can be retained when all three of the following criteria have been met (52): (1) rapid onset (less than 3 months) of at least four of the six major groups of symptoms, including (i) psychiatric symptoms or cognitive impairment, (ii) seizures, (iii) speech dysfunction, (iv) abnormal movements, (v) decreased consciousness, (vi) autonomic dysfunction or central hypoventilation; (2) positive CSF IgG anti-GluN1 antibodies; and (3) reasonable exclusion of other disorders. Importantly, anti-NMDAR encephalitis with OT had a more severe clinical presentation. Dai et al. reported that the incidence of fever, decreased consciousness, autonomic dysfunction, central hypoventilation and intensive unit care were significantly higher in patients with OT than in those without OT (53). Similarly, Jiang et al. observed that patients with OT required more frequently mechanical ventilation and intensive care, and had higher CSF antibody titers (54).

**Table 1:** Ovarian teratoma-related paraneoplastic syndromes.

Antibody	Antigen	Paraneoplastic neurological syndrome	References
NMDAR	GluN1	Encephalitis; meningoencephalitis; NMDAR encephalitis	(10, 15–39)
AMPAR	AMPAR	Limbic encephalitis	(40)
CASPR2	CASPR2	Limbic encephalitis	(9)
Yo	CDR2/CDR2L	Encephalomyelitis	(41)
Ri	Neuro-oncological ventral antigen	Limbic encephalitis	(42)
KLHL11	KLHL11	PCD, OMS, chronic psychosis or subacute encephalopathy	(43)
LUZP4		Encephalomyelitis	(44)
AQP4	AQP4	NMOSD	(7, 45, 46)
MOG	MOG	Recurrent ON, LETM, BSTE, encephalopathy	(6, 47, 48)

AMPAR, α-amino-3-hydroxy-5-methyl-4-isoxazole-propionicacid receptor; AQP4, Aquaporin-4; BSTE, brain stem encephalitis; CASPR2, contactin-associated protein-like 2; CDR2, cerebellar-degeneration-related protein 2; CDR2L, cerebellar degeneration-related protein 2-like; KLHL11, Kelch-like protein 11; LETM, longitudinally extensive transverse myelitis; LUZP4, leucine zipper 4; MOG, myelin oligodendrocyte glycoprotein; NMDAR, N-methyl-D-aspartate receptor; OMS, Opsoclonus myoclonus ataxia syndrome; ON, optic neuritis; PCD, progressive cerebellar syndrome.

Evidence of OT-related LE other than anti-NMDAR encephalitis is based on case series and reports in the context of the detection of a novel antibody. LE is characterized by the subacute progression (less than 3 months) of seizures, working memory deficits, or psychiatric symptoms (52). According to the updated diagnostic criteria of LE, antibody status is not needed, which could delay the diagnosis. However, the identification of autoimmune antibodies remains important, especially to identify the immunological subgroup of LE. Some of the onconeural and neuronal surface antibodies associated with OT-related LE, such as AMPAR, CASPR2 and Ri antibodies, have been reported recently (9, 40, 42).

### EM

The term encephalomyelitis should be used to describe those patients with clinical dysfunction at multiple sites of the nervous system, including LE, brainstem encephalitis, PCD, myelitis (anterior horn), sensory neuronopathy (dorsal root ganglia), and chronic gastrointestinal pseudo-obstruction (55). The evidence for paraneoplastic encephalomyelitis associated with OT is based on a small sample series or case reports. Some cases were combined with antineuronal antibodies (such as anti-Yo, anti-leucine zipper 4 [LUZP4], and anti-MOG antibodies) (6, 41, 44, 47, 48). some reports without screening antibodies, and in some cases, antibodies were not found, although extensive antibody screening was performed (56, 57).

### PCD and OMS

PCD manifests clinically as incoordination of movements (ataxia), balance and gait disturbances, speech disorder (dysarthria), and altered ocular movements (nystagmus, often in a downbeat form) (55). In a large retrospective review of 249 patients with teratoma-related encephalitis, 22 (4%) developed PCD (58). Another type of antibody found in PCD was kelch-like protein 11 (KLHL11), which is a new antibody that has been described by Mandel-Brehm et al. (59). Anti-KLHL11 antibody seropositive status suggests testicular germ-cell tumors or breast cancer. Surprisingly, a recent study found that among 16 female patients with positive KLHL11 antibodies, 10 (62.5%) patients had OTs. Two patients had PCD, 5 had anti-NMDAR encephalitis (with concurrent anti-NMDAR antibodies), 1 (10%) had opsoclonus-myoclonus and chronic psychosis, and 1 (10%) had subacute encephalopathy (43). However, a recent study found discordant results: in a French nationwide study, patients with KLHL11 antibodies showed a homogeneous clinical syndrome. All the patients were male, with a predominantly cerebellar/brainstem syndrome. Most of them had an associated testicular “burned-out” seminoma, and a large group of anti-NMDAR patients with associated OT were tested for KLHL11, with negative results (60).

OMS is a rare syndrome characterized by opsoclonus, which is spontaneous, irregular, multivectorial saccadic eye movements, along with focal or diffuse myoclonus and sometimes ataxia (55). In addition to KLHL antibodies as described above, one case of anti-NMDAR antibody-positive OT-associated OMS has been reported (29). In a large series with OMS (n = 114), 39% (45/114) of patients had paraneoplastic OMS. Among the patients with paraneoplastic OMS, 18% (8/45) had OTs, but all of them were NMDA antibody-negative (61).

### Nonclassical Syndromes

#### NMOSD

NMOSD is a relapsing inflammatory disease characterized by transverse myelitis and optic neuritis (ON) (52). The disease usually has seropositivity for AQP4-IgG in up to 80% of patients. In a recent review, Ikeguchi et al. analyzed 6 patients with AQP4-IgG–seropositive NMOSD associated with OT. All 6 patients presented with transverse myelitis, 2 with ON, and 5 with nausea and/or vomiting. In one case, anti-NMDAR encephalitis developed before NMOSD (7).

#### Autoimmune Glial Fibrillary Acidic Protein (GFAP) Astrocytopathy

Autoimmune GFAP astrocytopathy is a new autoimmune CNS disease diagnosable by GFAP-IgG testing in CSF (62). It may affect broad CNS regions, including subcortical white matter, basal ganglia, hypothalamus, cerebellum, brainstem, and spinal cord, although linear enhancement oriented radially to the ventricles is typical. Primary clinical manifestations of autoimmune GFAP astrocytopathy include fever, headache, abnormal vision, myelitis, encephalopathy, involuntary movement, and autonomic dysfunction (63). Approximately one in four individuals has a coexisting neoplasm, most commonly OT (63). In addition to GFAP-antibodies, the coexistence of anti-NMDAR and/or AQP4 antibodies was observed in more than half of the autoimmune GFAP astrocytopathy patients with OT (serologic complexity) (64).

## Neurological Examination

### Electroencephalogram (EEG)

In most patients with encephalitis, EEG may show a nonspecific pattern, including slow background activity, bilateral or unilateral temporal lobe epileptic discharges, and periodic lateralized epileptiform discharge. However, EEG may be helpful in patients with anti-NMDAR-encephalitis. It has been reported that one-third of anti-NMDAR-encephalitis patients showed extreme delta brush on EEG, although these EEG abnormalities are not specific for anti-NMDAR encephalitis (65).

### Cerebrospinal Fluid

In most cases of OT-related PNSs associated with cell-surface abs (e.g., antibodies against NMDARs and AMPARs), inflammatory changes and positive antibodies in CSF were observed (28, 40). In addition, 67% of cases with OT-related NMOSD showed mild to moderate CSF pleocytosis (7). Only a few OT-related PNSs cases associated with onconeuronal antibodies reported CSF analysis, two cases described elevated CSF protein and mild to moderate pleocytosis, and one case showed CSF oligoclonal bands positivity (41).

Interestingly, Jiang et al. observed higher CSF NMDAR antibody titers and increasing trends of cytokine/chemokines (including interleukin [IL]-10, IL-1α, tumor necrosis factor-α, granulocyte-macrophage colony-stimulating factor, and vascular endothelial growth factor A) in anti-NMDAR encephalitis patients with OT than in patients without tumors (54), which indicated an inflammatory process in the pathogenesis of paraneoplastic anti-NMDAR encephalitis. Although some experts proposed that the immune response of OT may trigger BBB dysfunction in PNSs, studies that measure brain barrier dysfunction (CSF and serum oligoclonal band, CSF albumin quotient, or intrathecal IgG synthesis rate) specifically for patients with OT-related PNSs are limited.

### Imaging Studies

Although neuroimaging studies are not key to establishing the diagnosis of PNS, they should be performed to exclude other diseases responsible for neurologic symptoms. In patients with OT-associated PNSs, MRI abnormalities are associated with PNSs type, e.g., PCD patients reveal cerebellar atrophy, anti-NMDAR encephalitis patients show abnormalities in various brain regions (such as the hippocampus, basal ganglia, brainstem, cerebral cortex, and insular regions), and NMOSD patients show characteristic dorsal brainstem lesions on MRI (7, 58, 66).

Importantly, if PNS is suspected, systemic imaging of the underlying tumor should be performed. Transvaginal ultrasound (may not be feasible in young patients) and/or MRI- elvis/abdomen is the investigation of choice for these patients. If negative, CT-chest searching for extra-pelvic teratomas is recommended. If initial examinations do not detect any neoplasm, it is recommended to repeat them every 6 months for 2 years (67, 68).

### Pathology

The histopathology of anti-NMDAR encephalitis associated OT has not been fully elucidated but limited studies have focused on alterations in immune cell populations and neuroglial tissues (**Table 2**) (16–26). In patients with anti-NMDAR encephalitis, the most common comorbid tumor type was unilateral MTs. Immature teratomas (ITs) represent only about 12-26% of all ovarian teratomas in patients with anti-NMDAR encephalitis (51). The diameter of the anti-NMDAR encephalitis associated OT is not very large (average 3.48 cm, ranging from 0.5 to 22 cm) when viewed grossly (16, 26). Anti-NMDAR encephalitis associated OT may not exhibit significant features after specimen dissection.

**Table 2:** Pathological characteristics in teratoma tissues in patients with paraneoplastic neurological syndromes.

Author (Ref.)	Abs	No.	Tumor size (cm) and laterality	Teratoma pathology	Inflammatory infiltrates	Nervous tissue component	Anomalous neurons	Expression	Comments
Dalmau et al. (16)	NMDAR	10	Mean size: 6.6 (range 1.5-22) Right side: 4 cases; Left side: 5 cases; Bilateral: 1 case	MT: 6 cases IT: 4 cases	NA	5/5	MAP2 positive immature neurons	NR2	Immature neurons present and NR2 detected on neuroglial tissue
Tüzün et al. (17)	NMDAR	2	Size: 3.5 and 1.5 respectively; Right side: 1 case; Left side: 1 case	MT: 2cases	CD3+ T cells, CD20+ B cells, CD79a+ B cells, CD68+ cells	2/2	NA	NR1/NR2	CD20+ B cells were more frequent in the PNSs group
Tabata E et al. (18)	NMDAR	3	NA	NA	CD3+ T cells, CD4+ T cells, CD8+ T cells, CD20+ B cells	3/3	NA	NR1/NR2	CD4+ T cells, CD8+ T cells and CD20+ B cells were more frequent in the PNSs group
Dabner et al. (19)	NMDAR	5	Mean size: 3.6 (range 0.7-9.5) Right side: 3 cases; Left side: 1 case; Bilateral: 1 case	MT: 4 cases IT: 1 case	CD3+ T cells, CD20+ B cells	4/5	neuroglial matrix (n = 4), and degenerative neuronal changes (n = 2)	NA	Degenerative changes present in the PNSs group
Day et al. (20)	NMDAR	5	Mean size: 3 (range 1.2-4.5)	MT: 4 cases IT: 1 case	CD3+ T cells, CD20+ B cells, CD79a+ B cells,	4/5	4/5 (gangliogliomas [n = 3] and ganglioneuroblastoma [n = 1])	NA	Abnormal neurons were more frequent in the PNSs group
Iemura et al. (21)	NMDAR	4	Median size: 5 (range 2.5-15) Right side: 2 cases; Left side: 2 cases	MT: 3 cases IT: 1 case	CD3+ T-cells and CD20+ B-cells	4/4	4/4 (mature neuroglial tissues, higher cell density, smaller volume and higher proliferative activity)	NR	NR expressing neurons were significantly densely aggregated and relatively smaller in size in the PNSs cases, and the Ki-67 labeling index of neuroglial cells with these neurons was significantly higher in the PNSs cases
Makuch et al. (22)	NMDAR	2	NA	NA	CD3+ T-cells, CD20+ B-cells and CD138 cells	NA	NA	NR1, NR2	Antibodies to NR1, NR2 detected in germinal center of lymphoid aggregates
Mitra et al. (23)	NMDAR	1	Size: 1.4; Right side	MT	CD45 T-cells	Positive	Immature neurons	NA	
Chefdeville et al. (25)	NMDAR	27	NA	MT: 24 cases IT: 3 cases	CD3+ T-cells and CD20+ B-cells	26/27	3/24 (1 oligodendroglioma-like, 1 ganglioma-like, 1 malignant glioma-like)	NR1	The massive infiltration by immune cells and particular glial features of its neuroglial component might be involved in triggering or sustaining the anti-tumor response associated with the autoimmune neurological disease
Nolan et al. (26)	NMDAR	12	Median size: 1.7 (range 0.5-5)	MT: 11 cases IT: 1 case	CD3+ T-cells and CD20+ B-cells	12/12	A relative paucity of mature neurons and a hypercellular astrocyte population	NA	NMDAR encephalitis related teratomas are characterized by co-localization of neuroglial tissue and lymphoid aggregates with germinal centers. The alterations in neuroglial cell populations are related to the pathogenesis of NMDAR encephalitis
Jiang et al. (54)	NMDAR	10	Ranged from 2.1 × 2 × 1.9 cm to 18.5 × 10.3 × 9 cm Right side: 2 cases; Left side: 8 cases	MT: 8 cases IT: 2 cases	CD3+ T-cells and CD20+ B-cells	10/10	Dysmorphic neurons with floating-frog neural elements	NR1, NR2A, NR2B and IgG	A cellular population of dysplastic neurons co-expressing NMDAR subunits were the potential source of autoantigens triggering anti-NMDARE
Xiao et al. (24)	NMDAR	3	Ranged from 7 × 6.5 × 4 cm to 9 × 9 × 5 cm Right side: 2 cases; Left side: 1 case		CD4+ T cells, CD8+ T cells, CD20+ B cells	3/3	Immature neurons	NR1, GFAP	Intense NR1 expression occurs in squamous epithelium near neuroglial tissue
Frasquet et al. (45)	AQP4	1	Right side	MT	CD3+ T cells, CD4+ T cells, CD20+ B cells and CD138+ cells	Positive	NA	AQP4	
Bernard-Valnet et al. (46)	AQP4	3	NA	NA	CD3+ T cells, CD4+ T cells, CD20+ B cells	3/3	3/3 neuroglial tissue revealed by a strong GFAP staining	AQP4	Teratomas revealed glial component strongly expressing AQP4 and closely localized to immune infiltrates
Ikeguchi et al. (7)	AQP4	1	Left side	MT	CD45RO+ or CD8+lymphocytes	Positive	Neuroglial tissue revealed by a strong GFAP staining	AQP4	The neural tissue exhibiting a GFAP+ glial component with AQP4 immunoreactivity
Wildemann et al. (6)	MOG	1	Size: 6 Right side	MT	CD4 + and CD8 + T cells, CD68 + as well as MHC II-expressing macrophages/activated microglia and CD1c + dendritic cells	Positive	Neuroectodermal tissue with expression of GFAP	MOG	Histopathology revealed neural tissue expressing MOG protein and accompanying immune cell infiltration within the teratoma, suggesting a possible paraneoplastic origin of MOG-EM

AQP4, Aquaporin-4; GFAP, glial fibrillary acidic protein; IT, immature teratoma; MT, mature teratoma; NA, not available; NMDAR, MOG, myelin oligodendrocyte glycoprotein; N-methyl-D-aspartate receptor; PCD, progressive cerebellar syndrome.

There are two main features of basic tissue staining for anti-NMDAR encephalitis-associated OT. First, nervous tissues were observed with higher frequencies in teratomas resected from anti-NMDAR encephalitis cases than controls (25). Moreover, aggregates of dysmorphic neurons (for example, floating-frog like dysplastic neurons, gangliogliomas, and ganglioneuroblastoma) were observed more frequently in anti-NMDAR encephalitis associated teratomas than sporadic teratomas (20, 26, 54). The association of these abnormal neurons within teratomas in the pathogenesis of anti-NMDAR encephalitis is unknown; one potential explanation is that these dysmorphic neurons may provide the nidus for an immune response triggering NMDAR antibody formation against tumor antigens (26). Second, the reduced density of mature neurons and degenerative changes in the neurons have been described in previous studies (19, 26). Nolan et al. suggested that these changes represent the result of sustained autoimmune injury to the neurons, containing a spectrum of damage ranging from degenerative changes to cell loss (26). Regarding the immunopathology of anti-NMDAR encephalitis associated OT, the striking particularity was that lymphoid B cells, T cells, and mature dendritic cells infiltration were predominant in the tumor tissue of the anti-NMDAR encephalitis group, and the inflammatory infiltrate was mostly adjacent to the receptor-positive nervous tissue or squamous epithelium in the teratoma tissue (18, 20, 24, 25, 54). Remarkably, CD20+ B cells were more frequent in anti-NMDAR encephalitis associated OT than in control OT, while CD8+ T cells were less frequent in anti-NMDAR encephalitis associated OT (25, 54). These immune cell populations suggest that humoral immunity plays an important role in anti-NMDAR encephalitis associated OT. In addition, the expression of the NR1, NR2A, and NR2B subunits by the teratoma nervous tissue was significantly more often glial in anti-NMDAR encephalitis associated OT than in control OT (18, 21, 25, 54). Specifically, immunofluorescence showed consistent colocalization of these NMDAR subunits with IgG, supporting the notion that dysmorphic neurons with coexpression of NR1/NR2A/NR2B and IgG in OT play a significant role in the pathogenesis of anti-NMDAR encephalitis (25, 54). Elevated proliferative activity was found in anti-NMDAR encephalitis-associated OT by using Ki-67 immunohistochemistry (24). Unfortunately, to date, no tumor markers are available to differentiate teratomas that may cause anti-NMDAR encephalitis. Last, although a study found significantly elevated levels of HLA-A and HLA-DBR1 in teratoma tissues from patients with anti-NMDAR encephalitis (69), the association of both alleles with disease susceptibility was weak and needs confirmation.

Due to the low incidence of OT-associated NMOSD, to date, there have been 5 reported cases describing pathologic findings of AQP4-IgG–seropositive NMOSD associated with OT (7, 45, 46). In all of these patients, teratomas revealed the expression of AQP4 and GFAP in areas containing neuroglial tissue. These areas were surrounded by intense inflammatory infiltrates, mainly composed of CD20 B+ cells. Only a few CD8+ T cells were observed in reported cases (7, 45, 46). These findings suggest that teratomas featuring AQP4-positive neural tissue accompanied by infiltrating lymphocytes contribute to NMOSD development. To our knowledge, only Wildemann et al. have previously described the pathological characteristics of MOG-EM associated teratoma and indicated the presence of MOG protein within the tumor (6). Analysis of tumor tissue from one Caucasian patient revealed teratoma-contained neuroectodermal tissue with the expression of GFAP. A detailed examination of the neuroectodermal tissue showed oligodendrocytes and axons with intermittent myelination, as demonstrated by the expression of oligodendroglial markers, including MOG. In addition, inflammatory infiltrates with CD4+ and CD8+ T lymphocytes were found. Additionally, CD68+ and MHC II-expressing macrophages/activated microglia and CD1c+ dendritic cells were observed in and immediately adjacent to the neuroectodermal tissue. Interestingly, CD20+ B cells and CD138-positive plasma cells were not observed in the teratoma, which was different from AQP4-IgG–seropositive OT and anti-NMDAR encephalitis associated OT (6).

Regardless of the type of PNSs, pathological features of OT include the expression of specific epitopes for autoantibodies and inflammatory infiltrate in the CNS-like tissue component. Whether mechanisms of mimicry toward protein expression or breakdown of immunologic tolerance are involved in OT-related PNSs is unknown. Dysmorphic neurons are currently only reported in anti-NMDAR encephalitis, and whether this abnormality exists in other types of PNSs needs to be further explored. The immunopathological findings of AQP4-IgG–seropositive OT were similar to those of anti-NMDAR encephalitis-associated OT. However, although it is difficult to draw definitive conclusions with only one case, we cannot rule out that OT may have different mechanisms for MOG-EM and anti-NMDAR encephalitis based on immunopathological findings. Given the small number of reported cases, our understanding of the pathological features of OT in PNSs is still limited.

### Treatments and Outcomes

The therapeutic methods of OT-related PNSs consist of tumor resection, immunotherapy, and symptom control. First-line immunotherapy includes corticosteroids, intravenous immunoglobulins, and plasma exchange. Second-line immunotherapy involves cyclophosphamide, rituximab, and mycophenolate mofetil (28). In addition, the treatment of immature teratoma requires chemotherapy according to its stage and postoperative histopathological grading. It has been reported that early tumor resection combined with immunotherapy markedly improves outcomes and prevents more severe neurological deterioration (15). Importantly, tumor resection has been reported to be more beneficial than immunotherapy in stopping neurological progression (70). In several cases with OT, severe neurological symptoms are reversible if the neoplasm is removed, and some do not even require additional immunotherapy. Previous studies suggest that critical systemic and neurological complications should not be considered contraindications for surgery (53). If teratoma resection is delayed, continued antigen presentation induces the production of long-lived plasma cells and increased antibody affinity, rendering the patient ineffective for late resection of the tumor and unresponsive to immunotherapy. Female patients with PNSs symptoms, especially those who do not respond to immunosuppressive therapy, should be checked for OT immediately. Prophylactic oophorectomy is not recommended in anti-NMDAR encephalitis without detectable OT. However, in selected patients with severe neurological involvement and proven lack of response to first- and second- line immunotherapies for more than 2-3 months, blind oophorectomy might be considered after carefully weighing the benefit/risk ratio of the procedure (2, 71).

Generally, in patients with PNSs, the response to therapy and prognosis depended on the underlying immune mechanism. Patients with antibodies against intracellular (onconeural) antigens (mediated by T cells) are more likely to have poor responses than patients with antibodies against surface or synaptic antigens (B cell mechanism) (72). The subgroup of patients with OT-associated anti-NMDAR encephalitis seems to have a good prognosis with partial or full recovery in about 80% of patients (50). Of note, a few cases experienced severe disability or even death. These patients often had treatment delays and serious complications, or their tumor tissue proved to be ITs. In addition, most of the patients with PNSs (including AQP4-IgG+ NMOSD, MOG-EM) had full recovery or almost full recovery after removal of teratoma and immunotherapy (6, 7). Improvement was observed in cases with OT-related anti-Ri encephalitis or anti-Yo encephalitis (41, 42). Due to the small number of patients that have been identified to date, further reports may be helpful to understand the therapeutic effect of OT-related PNSs.

Several studies have shown that early teratoma resection helps to reduce the recurrence of anti-NMDAR encephalitis (28, 53), and interestingly, previously published cases suggest that this condition seems to also be applicable for AQP4-IgG+ NMOSD and MOG-EM (6, 7). For example, it has been reported that about 30% of patients with AQP4-IgG+ NMOSD experience relapse despite treatment. However, in patients with AQP4-IgG+ NMOSD associated with OT, only 16.7% (1/6) of patients experienced relapse after removal of teratoma. Similarly, approximately 50% and 80% of patients who developed MOG-EM relapsed within the first 2 years and 5 years, respectively (73). However, in patients with MOG-EM associated with OT, none of them experienced recurrence after teratoma resection.

## Conclusions

Our knowledge of OT-related PNSs is still far from complete and remains to be investigated. First, studies have been conducted on large samples of patients with other tumors commonly associated with PNSs, such as small cell lung cancer and melanoma. However, there have been no large-scale prospective or retrospective studies investigating various autoantibodies in patients with OT. Our review suggests that it would be worthwhile to test for a panel of autoantibodies and not just specific autoantibodies against NMDAR-IgG in patients with OT presenting with PNSs. Second, there are few studies on the pathophysiology of OT-related PNSs. OT-related PNSs other than anti-NMDAR encephalitis are case reports. Therefore, whether there are pathophysiological differences in different types of OT-related PNSs is not yet known. Third, recent studies have reported that PNSs are not related to a particular histological tumor type but rather to a specific molecular signature of tumor cells. These studies suggested that tumor genetic alterations are the initial triggers. However, the genetic analysis in PNSs and OTs remains unknown.

Taken together, clinicians should be aware that in addition to anti-NMDAR encephalitis, OT may also be associated with other rare PNSs. These PNSs have to be recognized and managed effectively during the course of OT because they often respond well to tumor resection. Although significant progress has been made in understanding the pathogenesis and heterogeneity of diseases through molecular and immunopathology, further work is needed to investigate the pathophysiological process by which ovarian teratoma leads to PNSs.

## Author Contributions

JFL and MW drafted the manuscript. JW collected the data. JML conceptualized and designed the study and revised the manuscript. All authors contributed to the article and approved the submitted version.

## Funding

Supported by the National Natural Science Foundation of China (grants 82071459) and the Institute of Brain science and Brain-inspired technology of West China Hospital, Sichuan University (grants ZYJC21001).

## Conflict of Interest

The authors declare that the research was conducted in the absence of any commercial or financial relationships that could be construed as a potential conflict of interest.

## Publisher’s Note

All claims expressed in this article are solely those of the authors and do not necessarily represent those of their affiliated organizations, or those of the publisher, the editors and the reviewers. Any product that may be evaluated in this article, or claim that may be made by its manufacturer, is not guaranteed or endorsed by the publisher.
